# Prediction of 2-Year Incident Clinical Knee Osteoarthritis From Extensor Mechanism Mechanical Properties in Asymptomatic Community-Dwelling Older Adults: Prospective Cohort Study

**DOI:** 10.2196/84494

**Published:** 2026-09-24

**Authors:** Ui-jae Hwang, Zongpan Li, Peng Xia, Tianxiang Fan, Arnold Wong, Oh-yun Kwon, Siu-ngor Fu

**Affiliations:** 1 Department of Rehabilitation Sciences, The Hong Kong Polytechnic University, QT 508, The Hong Kong Polytechnic University, 11 Yuk Choi Road, Hung Hom, Kowloon, Hong Kong, China (Hong Kong), +852 2766 6726; 2 Department of Physical Therapy, College of Software and Digital Healthcare Convergence, Yonsei University - Mirae Campus, Wonju, Republic of Korea

**Keywords:** arthritis, elderly, machine learning, musculoskeletal, rehabilitation

## Abstract

**Background:**

Knee osteoarthritis (KOA) lacks disease-modifying treatment, making early identification of modifiable risk factors in asymptomatic older adults a priority. Patellar tendon stiffness (PTS) and knee extensor strength (KESt) may independently influence KOA development, yet their prognostic value in individuals free of knee symptoms is unestablished.

**Objective:**

This study aimed to determine whether baseline extensor mechanism mechanical properties predict 2-year incident clinical KOA in community-dwelling older adults who were asymptomatic at enrollment, and to quantify the relative contribution of each parameter.

**Methods:**

This 2-year prospective cohort study enrolled 116 community-dwelling older adults (220 knees) free of knee pain at baseline. Incident clinical KOA was diagnosed according to the American College of Rheumatology criteria. Prognostic performance was evaluated across 7 survival algorithms using 5-fold nested cross-validation with the Harrell C-index. Predictor importance was quantified using Shapley Additive Explanations (SHAP) values from the best-performing model, and absolute KOA risk was estimated across tertiles of the linear predictor score.

**Results:**

Over 2 years, 55 (25%) knees developed incident clinical KOA. PTS and KESt were consistently selected across all cross-validation folds, whereas quadriceps muscle passive stiffness was not selected in any fold. The gradient boosted survival model achieved the highest discrimination (C-index=0.720, 95% CI 0.686-0.754), significantly exceeding chance (*P*<.05). SHAP analysis identified KESt as the dominant predictor (mean |SHAP| 0.314, 95% CI 0.251-0.388), with PTS as the second most important predictor (0.166, 95% CI 0.128-0.200). Knees in the highest-risk tertile showed a 24-month KOA incidence of 38.4% (28/73), which was more than twice that in the lowest-risk tertile (13/74, 17.6%).

**Conclusions:**

PTS and KESt independently and consistently predicted 2-year incident clinical KOA in asymptomatic community-dwelling older adults. Extensor mechanism mechanical profiling may identify individuals at elevated KOA risk prior to symptom onset, although these exploratory findings require confirmation in larger cohorts before KESt and PTS can be considered for screening or preventive intervention.

## Introduction

Knee osteoarthritis (KOA) is among the most prevalent musculoskeletal conditions affecting older adults worldwide, with an estimated 595 million individuals affected globally in 2020 and projections indicating a 74.9% increase by 2050 [1]. This condition is associated with substantial functional limitation and currently lacks disease-modifying pharmacological treatment [2], making early identification of modifiable prognostic markers in asymptomatic community-dwelling older adults a research priority. Among the candidate domains, biomechanical factors governing tibiofemoral load distribution are among the most modifiable determinants of KOA onset, and the extensor mechanism represents a primary target given its central role in dynamic knee joint stabilization during weight-bearing activities [3,4].

The extensor mechanism of the knee, comprising the quadriceps muscle group and the patellar tendon, functions to extend the tibiofemoral joint through coordinated muscular force transmission to the tibial tuberosity [5]. Although historically conceptualized as a simple pulley system, biomechanical evidence indicates that it operates as a mechanically complex unit in which the quadriceps musculature and patellar tendon function interdependently [3,5,6]. Three mechanical properties within this integrated system have each been independently supported by prospective evidence as contributors to KOA-related pathology: knee extensor strength (KESt), quadriceps muscle passive stiffness (QMPS), and patellar tendon stiffness (PTS).

KESt is a modifiable risk factor for KOA development. A systematic review and meta-analysis of 46,819 participants demonstrated that quadriceps weakness is a robust prospective risk factor for KOA [7], and a meta-analysis of 15 longitudinal studies found that lower KESt was associated with symptomatic progression in established KOA [8]. Reduced KESt has been associated with increased peak tibiofemoral contact forces and impaired joint stabilization during dynamic loading, which may contribute to articular cartilage degeneration [9-11]. Increased QMPS is a recognized consequence of aging arising from changes in viscoelastic tissue properties and intramuscular connective tissue deposition [12,13], and prospective evidence demonstrates that baseline QMPS is independently associated with incident clinical KOA over 12 to 24 months in community-dwelling older adults [14,15]. Although PTS generally declines with age due to structural degeneration [16], substantial interindividual variation persists even among asymptomatic older adults [17]. This interindividual variation in PTS among asymptomatic older adults may reflect subclinical differences in tendon mechanical integrity prior to symptom onset, and reduced patellar tendon elasticity has been observed in individuals with established KOA compared to asymptomatic controls [18].

Although these 3 parameters have each been examined as individual predictors in separate prospective studies, no study has simultaneously evaluated their combined predictive value, and existing approaches have relied predominantly on conventional regression methods that assume prespecified linear relationships and may not adequately characterize the nonlinear interactions likely to be present among mechanically interdependent predictors [19,20]. Unlike conventional Cox regression, which requires prespecified functional forms for each predictor, ensemble-based machine learning survival algorithms adapt to the structure of the data without assumptions about linearity or additivity and are capable of detecting complex interactions among simultaneously entered predictors. When applied within nested cross-validation frameworks with permutation-based significance testing, these methods provide discrimination estimates that are less susceptible to optimism bias than conventional regression concordance statistics [19], and Shapley Additive Explanations (SHAP) values further allow the relative contribution of each predictor to be quantified within the context of the full predictor set [21].

The purpose of this study was to determine whether baseline KESt, QMPS, and PTS, assessed in community-dwelling older adults who were asymptomatic at enrollment, predict the 2-year incidence of clinical KOA. Secondary aims were to quantify the relative prognostic contribution of each mechanical parameter through SHAP decomposition and to estimate absolute clinical KOA risk across predicted risk tertiles. We hypothesized that at least 1 of the fitted survival models would achieve a C-index of ≥0.70, indicating acceptable discrimination for the 2-year incidence of clinical KOA.

## Methods

### Study Design and Participants

This study used data from a 2-year prospective cohort study conducted between November 2018 and October 2019. Community-dwelling older adults aged 60 to 80 years who were able to walk 10 meters independently were recruited from local communities. Individuals were excluded if they reported hip, knee, or ankle pain at baseline, had a history of knee injury requiring medical treatment, demonstrated cognitive impairment based on the Montreal Cognitive Assessment, or had a diagnosed neurological condition. Of 225 participants (450 knees) assessed for eligibility, 142 (63%) participants (271/450, 60% knees) met the inclusion criteria. Following the exclusion of knees with missing follow-up data for clinical KOA, 49% (220/450) of knees from 52% (116/225) of participants were included in the present analysis.

A formal a priori sample size calculation was not performed, as this study used data from an existing prospective cohort. The study sample comprised 55 incident clinical KOA events, corresponding to an events-per-variable (EPV) ratio of 9.2 for the primary model. Although this was marginally below the commonly cited threshold of 10, simulation evidence demonstrates that EPV values of 5 to 10 produce acceptable parameter estimation when predictors are continuous and prespecified on theoretical grounds rather than identified through data-driven exploration [22], both of which apply to the present predictor set. Because knees were clustered within participants, we additionally quantified the effective information size. The 55 knee-level events occurred in 31% (36/116) of participants (17/36, 47% unilateral and 19/36, 53% bilateral). The within-participant correlation of the clinical KOA outcome between the 2 knees of a participant was high (intraclass correlation coefficient [ICC] 0.91), corresponding to a design effect of 1.82 and a clustering-adjusted effective EPV of 5.0, which we regarded as the lower bound for interpreting model stability.

### Ethical Considerations

The study was approved by the Human Subjects Ethics Sub-committee of the Hong Kong Polytechnic University (HSEARS20180110001), and all participants provided written informed consent prior to participation in accordance with the Declaration of Helsinki.

### Baseline Predictor Measurements

The primary predictor set comprised 3 extensor mechanism mechanical properties, each selected on the basis of independent prospective evidence linking it to KOA-related pathology: PTS, QMPS, and KESt [7,14,15,18]. Age, sex, and BMI were included as covariates. Full measurement protocols, equipment specifications, and reliability data are provided in Multimedia Appendix 1.

PTS (kPa) was measured using shear-wave ultrasound elastography (Aixplorer version 4.2; Hologic Inc), with participants positioned supine and the knee flexed to 30° [23]. After 5 minutes of rest to unload tendon tension, the patellar tendon was located longitudinally using B-mode ultrasonography. Shear elastic modulus was calculated using the equation E=3ρc², where ρ is tissue density (1000 kg/m³), and c is shear wave velocity. Three measurements were obtained and averaged. Intrarater reliability was excellent (ICC 0.98) [23].

QMPS (kPa) was defined as the unweighted mean shear modulus of the rectus femoris, vastus lateralis, and vastus medialis, each measured by shear-wave ultrasound elastography at standardized anatomical sites with the knee at 60° flexion [15]. This composite formulation was adopted to reduce the predictor count relative to the number of outcome events, thereby improving the EPV ratio. Intercorrelations among the 3 component muscles were moderate (*r*=0.34-0.47), supporting their aggregation into a single composite index. Intrarater reliability was excellent across all sites (ICC 0.89-0.94) [15].

KESt (Nm/kg) was assessed as isokinetic peak torque at 60°/s using an isokinetic dynamometer (Cybex Co), with participants seated and the dynamometer axis aligned with the lateral femoral epicondyle [10]. Peak torque values from the middle 3 of 5 maximal concentric contractions were averaged and normalized to body mass. All 3 mechanical predictors were measured separately on the left and right limbs, and each knee’s predictor values were linked to the incident clinical KOA outcome of the corresponding knee, so that every knee contributed a self-contained set of limb-specific predictors and an outcome.

### Outcome: Incident Clinical KOA

All participants were invited for in-person clinical examination between 12 and 24 months after baseline assessment, with the specific timing of each visit recorded for time-to-event analysis. Clinical examinations were performed by a single experienced physiotherapist blinded to all baseline measurements. Clinical KOA was diagnosed according to the American College of Rheumatology (ACR) clinical classification criteria [15,24,25]. Given that all participants were aged >60 years, a knee was classified as having clinical KOA if it demonstrated knee pain with an intensity of Visual Analog Scale (VAS) ≥3/10 during the examination and any 2 of the following 5 features: morning stiffness lasting <30 minutes, crepitus on active joint motion, bony tenderness on palpation, bony enlargement on inspection or palpation, or absence of palpable warmth of the synovium [15]. Knees that did not meet these criteria at the time of examination were recorded as event-free, with follow-up time defined as the time from baseline to the clinical examination visit. Pain intensity and each physical examination finding were assessed and recorded separately for the left and right knee, and the classification criteria were applied to each knee independently, so that a participant could be classified as having incident clinical KOA in 1 knee, both knees, or neither.

### Machine Learning Approaches and Statistical Analysis

All analyses were performed using R (version 4.5.2; R Foundation for Statistical Computing). Missing predictor data (maximum 2.73% per variable) were handled using multiple imputation by chained equations (m=20 datasets, predictive mean matching), with estimates pooled using Rubin rules [26]. The primary predictor set comprised PTS, QMPS, and KESt, adjusted for age, sex, and BMI, producing an EPV ratio of 9.2, considered acceptable for prespecified predictors with strong theoretical priors [22].

Seven survival prediction algorithms were compared: cluster-robust Cox proportional hazards regression (Cox_CR) SEs as the statistical reference, accounting for within-person correlation between bilateral knees [27]; Least Absolute Shrinkage and Selection Operator (LASSO)–penalized Cox regression (LASSO_Cox) [28]; ElasticNet-penalized Cox regression (ElasticNet_Cox) [29]; random survival forest (RSF); gradient boosting survival (GBS) and componentwise LASSO GBS (LGBS) [30]; and a regression-type survival support vector machine (SurvSVM) with a radial basis function kernel [31]. All hyperparameters were optimized within each outer training fold via dedicated inner cross-validation, ensuring that no test-fold observations influenced parameter selection (Multimedia Appendix 2). Prior to hyperparameter tuning, a LASSO-penalized Cox model with 5-fold inner cross-validation (lambda.1se criterion) was applied within each outer training fold to select a parsimonious feature subset, ensuring that variable selection was entirely independent of test-fold observations.

All algorithms were evaluated using 5-fold nested cross-validation, with folds stratified at the participant level by event status to prevent data leakage from bilateral knee observations (Multimedia Appendix 3). Predictor subsets were identified within each outer fold via inner LASSO_Cox, and the selected predictors were then passed to all algorithms. Discrimination was quantified using Harrell C-index (mean across 5 folds; 95% CIs from fold-level SEs), and a participant-level permutation test (999 permutations) was used to confirm that the best-performing model exceeded chance.

Calibration and predictor importance were assessed in a participant-level held-out 20% test set. Calibration at 24 months was evaluated by comparing Kaplan-Meier–observed with model-predicted clinical KOA probabilities across 3 risk tertiles, supplemented by the inverse probability of censoring-weighted Brier score [32]. Predictor contributions were quantified using SHAP values with a Monte Carlo approximation, and directionality was confirmed from hazard ratios (HRs) estimated in a cluster-robust Cox model on the same training partition. The study sample was additionally stratified into low, intermediate, and high clinical KOA risk tertiles by linear predictor score, with 24-month Kaplan-Meier incidence estimated for each tertile. Sex-stratified C-indices were reported with bootstrap 95% CIs, and a sex-by-predictor interaction term was tested in a cluster-robust Cox model. Within-participant correlation between bilateral knees was handled consistently across all components of the analysis. Both knees of a participant were always kept together within the same partition: the nested cross-validation folds and the final 80-20 held-out split were assigned at the participant level, so that no participant contributed knees to both the training and evaluation sets. Consequently, all machine learning algorithms, the C-index, calibration, and the inverse probability of censoring-weighted Brier score were trained and evaluated on participant-disjoint partitions, and SHAP values were computed only on the held-out test set. In the permutation test, outcome labels were permuted at the participant level so that both knees of a participant received the same permuted status, preserving the clustered data structure under the null. The Cox reference model additionally used cluster-robust SEs. As a sensitivity analysis, we repeated the modeling with each participant contributing a single observation, collapsing each participant to their more affected knee (the knee with incident clinical KOA, or, when both knees were concordant, the knee with the higher PTS). We then compared the discrimination of this participant-level model with that of the primary knee-level model on a common participant-level held-out test set and tested the difference in C-index using a bootstrap resampling procedure at the participant level.

## Results

### Participant Characteristics

**Figure 1.**
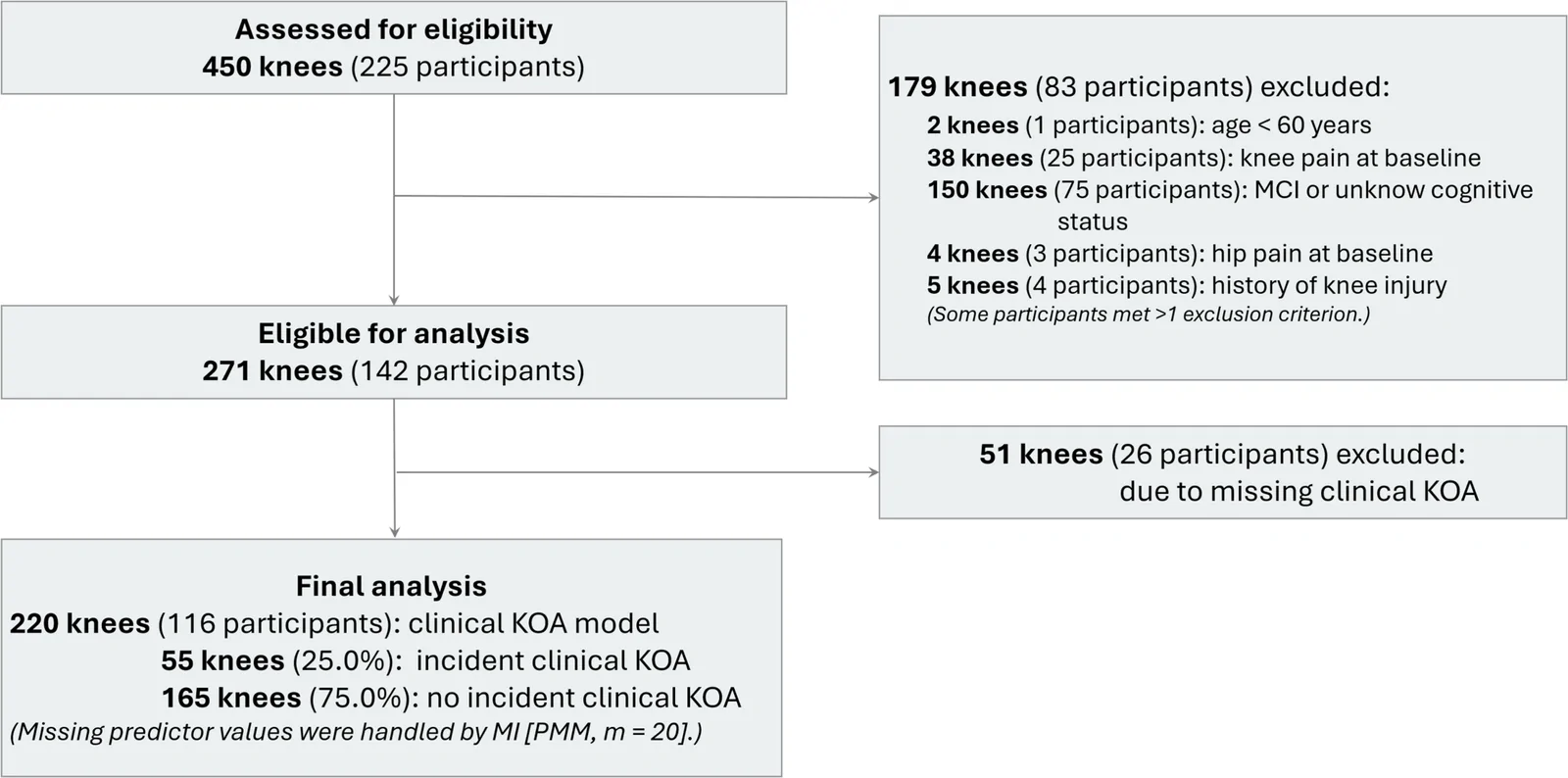
Participant flow diagram. A total of 450 knees (225 participants) were screened for eligibility. Following application of the exclusion criteria, 60% (271/450) of knees (142/225, 63% participants) were eligible for analysis. After exclusion of knees with missing follow-up data for the clinical KOA outcome (51/450, 11% knees from 26/225, 12% participants), 49% (220/450) of knees (116/225, 52% participants) were included in the final analytic sample. Missing predictor data (maximum 2.73% per variable) were handled using multiple imputation by chained equations. KOA: knee osteoarthritis; MCI: mild cognitive impairment; MI: multiple imputation; PMM: predictive mean matching.

The analytic sample comprised 220 knees from 116 participants, of whom 55 (25%) knees developed incident clinical KOA over the 2-year follow-up (Figure 1). Missing predictor data were minimal (maximum 2.73% per variable; Multimedia Appendix 4) and handled using multiple imputation. Groups did not differ significantly in age, sex, or BMI. At baseline, knees that subsequently developed clinical KOA showed significantly higher PTS (51.1 vs 40.0 kPa; *P*=.046) and lower KESt (1.14 vs 1.29 Nm/kg; *P*=.01) than knees that did not develop clinical KOA. QMPS did not differ between groups (*P*=.54; Table 1). Intercomponent correlations among the 3 quadriceps stiffness measures were moderate (Spearman *r*=0.344-0.472), supporting their aggregation into a single composite variable (Multimedia Appendix 5). Because clinical KOA is diagnosed at the level of the individual knee and the 2 knees of a participant can differ in both their mechanical properties and their outcome, each knee was treated as a separate observational unit, and incident clinical KOA was ascertained and counted per knee. The 55 incident knee-level events occurred in 36 participants, 47% (17/36) developed clinical KOA in 1 knee (unilateral) and 53% (19/36) in both knees (bilateral); within-participant correlation between paired knees was accounted for in all analyses.

**Table 1.** Baseline characteristics of the analytic sample stratified by 2-year incident clinical knee osteoarthritis (KOA; N=220 knees from 116 participants)^a^.

Characteristics	No KOA (n=165)	KOA (n=55)	*P* value^b^	Effect size (95% CI)^c^
Age (years), mean (SD)	65.7 (3.6)	66.0 (3.6)	.67	0.07 (−0.24 to 0.37)
Sex: female, n (%)	102 (62)	35 (64)	.94	0.01 (1.08; 0.57 to 2.04)
BMI (kg/m^2^), mean (SD)	23.5 (3.2)	23.9 (3.1)	.46	0.11 (−0.19 to 0.42)
Quadriceps composite stiffness (kPa), mean (SD)	7.21 (1.77)	7.40 (2.04)	.54	0.10 (−0.20 to 0.41)
Patellar tendon stiffness (kPa), mean (SD)	40.0 (22.3)	51.1 (38.2)	.046^d^	0.41 (0.10 to 0.71)
Knee extension strength (Nm/kg), mean (SD)	1.29 (0.32)	1.14 (0.36)	.01^d^	−0.43 (−0.74 to −0.12)

a Both knees from each participant were included as independent observational units; 116 participants contributed 220 knees. Demographic variables reflect participant-level data, whereas mechanical variables reflect knee-level data.

b Between-group comparisons were performed using an independent *t* test for continuous variables and a chi-square test for categorical variables.

c Effect size is Cohen *d *with 95% CI for continuous variables; positive values indicate a higher mean in the KOA group. For sex, Cramer V (odds ratio; 95% CI) is reported. Standardized effect sizes with CIs are reported in preference to post hoc statistical power.

d *P*<.05.

### Feature Selection

LASSO-penalized inner Cox regression consistently selected PTS and KESt in all 5 outer cross-validation folds (5/5 each). QMPS was not selected in any fold (0/5). In 1 fold, age and sex were additionally selected alongside the 2 mechanical predictors (Multimedia Appendix 6).

### Model Discrimination

**Figure 2.**
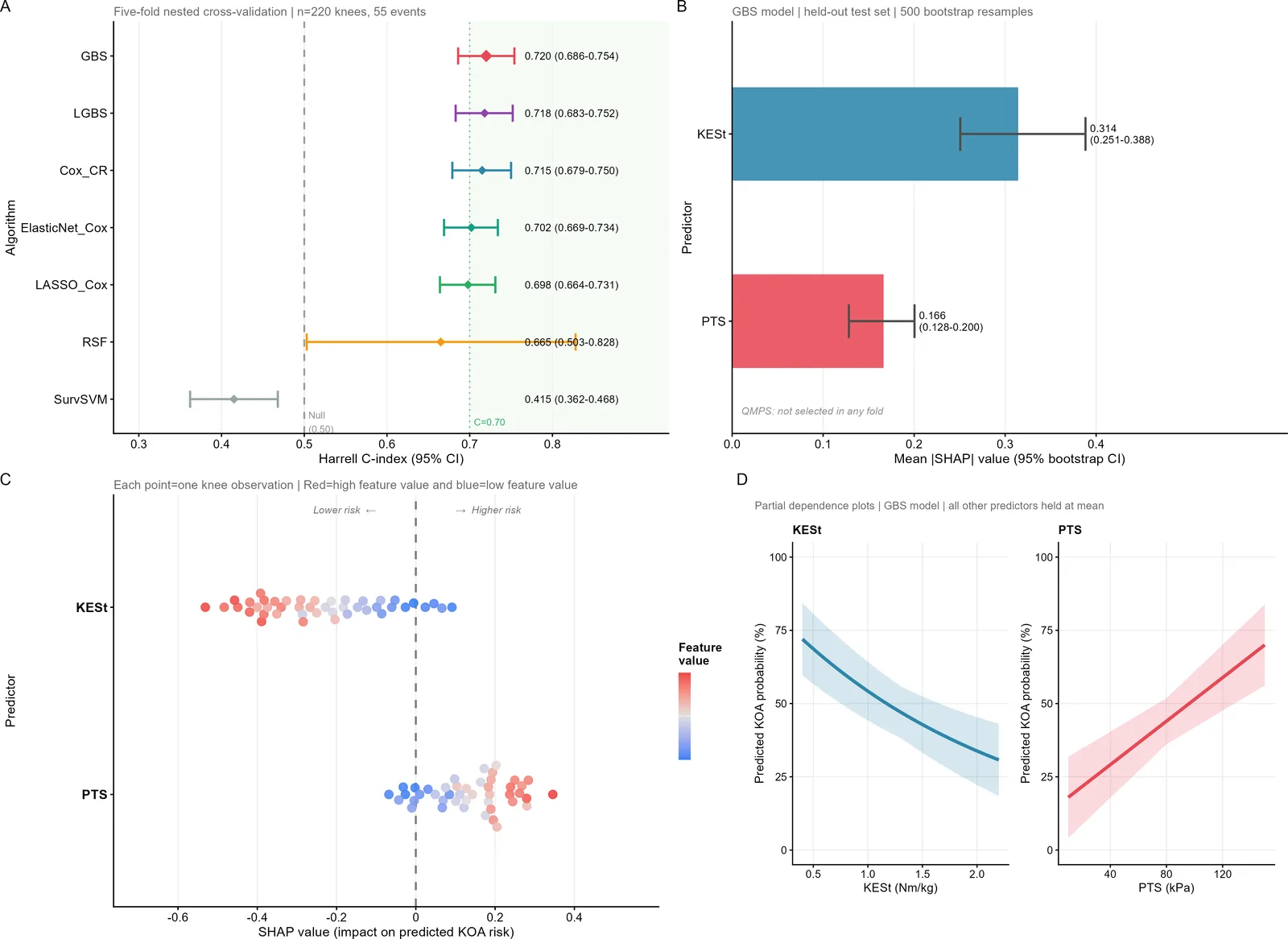
Machine learning model performance and predictor importance for clinical knee osteoarthritis (KOA) prediction. (A) Forest plot of Harrell C-index values for 7 algorithms (5-fold nested cross-validation; n=220 knees, 55 events). Diamond points and bars represent the mean C-index and 95% CI across folds; the dashed line represents the null model (C=0.50). Gradient boosting survival (GBS) achieved the best discrimination (C=0.720, 95% CI 0.686-0.754; permutation *P*<.01). (B) Mean absolute Shapley Additive Explanations (SHAP) values for the GBS model in the held-out test set; error bars represent the bootstrap 95% CI. Knee extensor strength (KESt) was the dominant predictor (0.314, 95% CI 0.251-0.388), followed by patellar tendon stiffness (PTS), ranked second (0.166, 95% CI 0.128-0.200). (C) SHAP beeswarm plot; each point represents 1 knee, with color indicating feature value (red=high and blue=low). (D) Partial dependence plots for KESt (left) and PTS (right); shaded bands represent the 95% CI. Cox_CR: cluster-robust Cox proportional hazards regression; LGBS: LASSO gradient boosting survival; RSF: random survival forest; SurvSVM: survival support vector machine.

All penalized and ensemble algorithms exceeded the null model (C=0.500; Figure 2). GBS achieved the highest discrimination (C=0.720, 95% CI 0.686-0.754), followed closely by LGBS (C=0.718, 95% CI 0.683-0.752) and Cox_CR (C=0.715, 95% CI 0.679-0.750; Table 2). RSF showed comparable mean discrimination (C=0.665) but substantially greater fold-level variability (SD 0.185) than all other algorithms (Multimedia Appendix 7). SurvSVM performed below chance level (C=0.415). Both GBS and Cox_CR significantly exceeded chance-level discrimination by permutation test (*P*<.01; Multimedia Appendix 8).

**Table 2.** Discrimination performance (Harrell C-index) of 7 survival prediction algorithms evaluated using 5-fold nested cross-validation (n=220 knees, 55 events)^a^.

Algorithms	C-index, mean (SD)		95% CI
Cluster-robust Cox proportional hazards regression	0.715 (0.040)		0.679-0.750
LASSO^b^-penalized Cox regression	0.698 (0.038)		0.664-0.731
ElasticNet-penalized Cox regression	0.702 (0.037)		0.669-0.734
Random survival forest	0.665 (0.185)		0.503-0.828
*Gradient boosting survival^c^*	*0.720 (0.039)*		*0.686-0.754*
Componentwise LASSO gradient boosting survival	0.718 (0.040)		0.683-0.752
Survival support vector machine	0.415 (0.060)		0.362-0.468

a C-indices are means across 5 outer folds; 95% CI derived from fold-level SEs. Folds were stratified at the participant level by event status.

b LASSO: Least Absolute Shrinkage and Selection Operator.

c Italicized text represents the best-performing algorithm.

### Predictor Importance and Directionality

SHAP analysis of the GBS model in the held-out test set identified KESt as the dominant predictor (mean |SHAP|=0.314, 95% CI 0.251-0.388), with higher strength consistently associated with lower clinical KOA risk across individual observations (Figure 2). PTS was the second most important contributor (mean |SHAP|=0.166, 95% CI 0.128-0.200), with higher stiffness associated with higher clinical KOA risk. Partial dependence plots confirmed monotonic dose-response relationships for both predictors in the expected directions (Figure 2). These directional findings were further confirmed by cluster-robust Cox HRs estimated on the same training partition: HR of 0.221 (95% CI 0.081-0.601; *P*=.02) for KESt and HR of 1.012 (95% CI 1.002-1.021; *P*=.049) for PTS (Table 3).

**Table 3.** Feature selection frequency, Shapley Additive Explanations (SHAP) predictor importance, and directionality for the gradient boosting survival model^a^.

Predictors	Selection frequency	SHAP rank	Mean |SHAP| (95% CI)^b^	Hazard ratio (95% CI)^c^	Direction
Knee extension strength (Nm/kg)	5/5	1	0.314 (0.251-0.388)	0.221 (0.081-0.601)^d^	Higher to lower risk
Patellar tendon stiffness (kPa)	5/5	2	0.166 (0.128-0.200)	1.012 (1.002-1.021)^d^	Higher to higher risk
Quadriceps composite stiffness (kPa)	0/5	—^e^	—	—	Not selected

a Feature selection was performed via Least Absolute Shrinkage and Selection Operator–penalized Cox regression (lambda.1se) independently within each outer training fold.

b Mean absolute SHAP values and 95% CI were derived from 500 bootstrap resamples (200 Monte Carlo simulations).

c Hazard ratios and 95% CI were estimated from a cluster-robust Cox model on the same training partition.

d *P*<.05.

e Not applicable (because the predictor was not selected in any fold).

### Absolute Risk Stratification and Calibration

**Figure 3.**
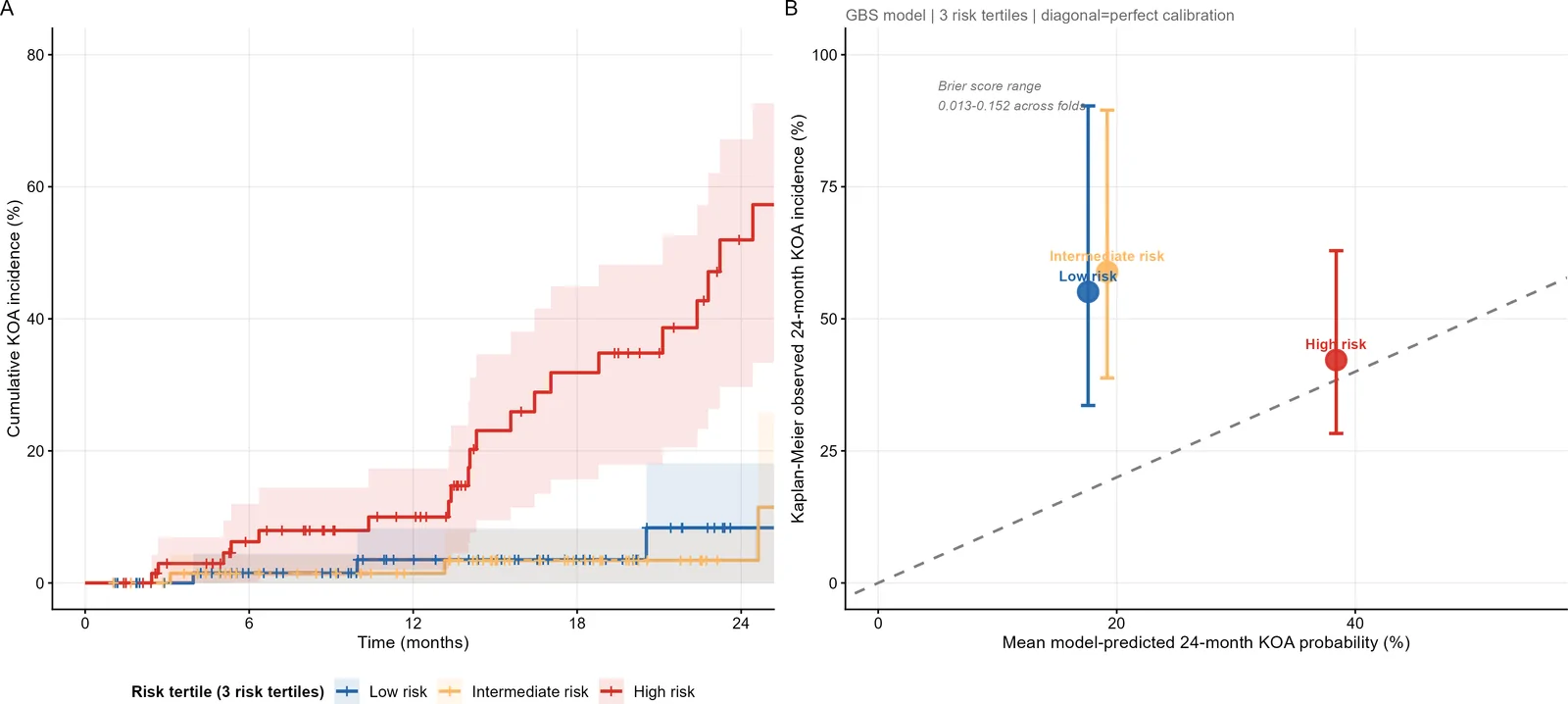
Absolute risk stratification and model calibration. (A) Kaplan-Meier knee osteoarthritis (KOA)–free survival curves stratified by gradient boosting survival (GBS) linear predictor risk tertile (n=220 knees, 116 participants). Low-risk tertile (n=74 knees, 47 participants, 13 events), intermediate-risk tertile (n=73 knees, 57 participants, 14 events), and high-risk tertile (n=73 knees, 48 participants, 28 events). The 24-month KOA incidence was 17.6% (13/74), 19.2% (14/73), and 38.4% (28/73) in the low-, intermediate-, and high-risk tertiles, respectively. Shaded areas represent the 95% CI. Risk tables below the panel indicate the number of knees remaining at risk at each time point. (B) Calibration plot at 24 months comparing mean model-predicted KOA probability with Kaplan-Meier–observed incidence across the 3 risk tertiles. Error bars represent 95% CI. The diagonal dashed line represents perfect calibration. The inverse probability of censoring-weighted Brier score at 24 months ranged from 0.013 to 0.152 across folds.

The inverse probability of censoring-weighted Brier score at 24 months ranged from 0.013 to 0.152 across folds (Multimedia Appendix 9). Although fold-level variability in the Brier score was observed, mean model-predicted probabilities were broadly consistent with Kaplan-Meier–observed incidence across risk tertiles, indicating acceptable calibration at the group level. Stratification into low, intermediate, and high clinical KOA risk tertiles by GBS linear predictor score generated 24-month clinical KOA incidence rates of 17.6% (13/74), 19.2% (14/73), and 38.4% (28/73), respectively, with the high-risk group showing more than twice the event rate of the low-risk group (Figure 3, Multimedia Appendix 10). Model-predicted probabilities were broadly consistent with Kaplan-Meier–observed incidence across tertiles (Figure 3, Multimedia Appendix 11).

### Sensitivity Analysis

The GBS model demonstrated consistent discrimination in male (C=0.727, 95% CI 0.564-0.835) and female (C=0.748, 95% CI 0.663-0.826) subgroups. A formal sex-by-predictor interaction term in a cluster-robust Cox model was nonsignificant (*P*=.49), indicating no meaningful effect modification by sex (Multimedia Appendix 12, Multimedia Appendix 13). In the participant-level sensitivity analysis, in which each participant contributed a single knee, discrimination on the common held-out test set was similar to that of the primary knee-level model (C=0.685 vs 0.731). The difference in C-index was not statistically significant (difference=0.046, 95% CI −0.096 to 0.162; *P*=.50). The direction of the predictor associations was preserved, with higher PTS associated with higher risk (HR 1.013; *P*=.01) and higher KESt associated with lower risk (HR 0.244; *P*=.02). These results indicate that the main findings are not an artifact of treating the 2 knees of a participant as independent observations.

## Discussion

### Principal Findings

In this 2-year prospective cohort study of community-dwelling older adults who were asymptomatic at enrollment, KESt and PTS were consistently identified as prognostic predictors of incident clinical KOA across all cross-validation folds, with the prespecified discrimination threshold of C-index ≥0.70 being met. To our knowledge, prospective time-to-event evidence linking higher PTS to incident clinical KOA has not previously been reported, and the present findings extend the existing literature by simultaneously quantifying the relative prognostic contributions of KESt and PTS within the same predictor set.

KESt emerged as the dominant predictor across all model interpretability metrics, with higher strength associated with lower KOA risk. This direction is consistent with prospective evidence that quadriceps weakness predicts KOA onset and progression [7,8,11] and extends those findings by quantifying the contribution of KESt within a simultaneously entered extensor mechanism predictor set. The cluster-robust Cox HR on the training partition (HR 0.221, 95% CI 0.081-0.601) was consistent in direction with previous studies [33,34]. Partial dependence analysis confirmed a monotonic dose-response relationship between KESt and predicted KOA risk, indicating a graded rather than threshold effect, which supports the inference that KESt may be a modifiable factor worth investigating as a potential target for preventive intervention in asymptomatic older adults at elevated KOA risk.

Greater baseline PTS was independently associated with a higher 2-year incidence of clinical KOA. A previous study examining PTS in participants with KOA was limited to cross-sectional designs, which cannot establish whether altered tendon mechanical properties precede or follow structural joint changes [18]. The present findings address this gap by demonstrating that PTS measured in asymptomatic older adults prior to any KOA diagnosis carries independent prognostic information over a 2-year follow-up period. From a mechanical perspective, a stiffer patellar tendon has been proposed to absorb less of the peak force produced during dynamic loading, resulting in higher tibial contact forces during locomotion [16]. Sustained elevation of tibiofemoral contact stress is a recognized contributor to articular cartilage degeneration, and patellar tendon enthesis abnormalities assessed on magnetic resonance imaging (MRI) have been shown to be associated with both knee pain and structural abnormalities in community-dwelling older adults [35], indicating that mechanical alterations at the tendon-bone interface may contribute to the pathological process leading to clinical KOA. Structural patellar tendon abnormalities are prevalent even in asymptomatic individuals, estimated at 22% to 36% across imaging modalities and populations [36], and the present findings suggest that mechanical stiffness carries prognostic information that is not fully explained by structural abnormality alone. It should be noted that shear-wave elastography provides a measure of tissue shear modulus as an index of passive mechanical stiffness, which is distinct from direct assessment of tendon viscoelasticity, thickness, or tensile properties. Future studies incorporating multiple indices of patellar tendon mechanical behavior may more comprehensively characterize the relationship between tendon properties and KOA risk. Whether age-related changes in tendon elasticity, cumulative mechanical loading history, or physical activity levels contribute to the elevation in PTS that preceded KOA onset in the present cohort warrants investigation in future studies with longer follow-up and serial tendon assessments. In contrast, QMPS was not selected in any cross-validation fold, suggesting that passive muscle stiffness at rest may lack independent prognostic value once active force-generating capacity and tendon mechanical properties are simultaneously accounted for. This pattern indicates that the prognostic signal within the extensor mechanism is carried primarily by its active and structural components rather than by passive muscle stiffness in isolation.

The application of survival analysis with predictor importance decomposition to this prediction problem produced 3 findings of clinical relevance. First, the consistency of predictor selection across all 5 cross-validation folds indicates that PTS and KESt carry a stable prognostic signal independent of data partition. Second, SHAP-based decomposition quantified the relative contribution of each predictor without imposing prespecified functional forms, identifying KESt as contributing approximately twice the predictive weight of PTS. Partial dependence analysis confirmed that both relationships were monotonic, indicating that the associations are adequately characterized by graded, directional effects rather than nonlinear interactions. The primary value of the analytical approach in the present study lies in its capacity to simultaneously rank predictor contributions and verify functional form assumptions without requiring a priori specification. Finally, risk stratification by linear predictor score identified a high-risk tertile with a 24-month KOA incidence more than twice that of the low-risk tertile. This gradient suggests that older adults with concurrently low KESt and high PTS constitute an identifiable subgroup for whom targeted preventive strategies, including quadriceps strengthening and tendon loading programs, may warrant priority consideration, pending confirmation in prospective interventional designs.

Several limitations of this study should be acknowledged. First, clinical KOA was diagnosed according to ACR clinical classification criteria without radiographic confirmation, which precludes direct comparison with studies using Kellgren-Lawrence grading as the outcome and may have introduced misclassification at the diagnostic boundary. Second, the study was conducted in a single community-based cohort, and external validation in an independent cohort is required before the risk stratification thresholds reported here can be considered for clinical application. Third, the 12-to-24–month follow-up window may be insufficient to capture the full spectrum of KOA incidence in an asymptomatic older population, given the typically prolonged natural history of the disease. Fourth, PTS was measured at 30° knee flexion with the tendon in a partially unloaded state, which may not fully reflect the functional stiffness experienced during weight-bearing activities. Finally, the nominal EPV ratio of 9.2 was calculated from the 55 knee-level events, marginally below the conventional threshold of 10, which may have introduced instability in the penalized regression estimates, particularly in folds with fewer events. Moreover, because the 55 knee-level events were clustered within 36 participants, the effective number of independent outcome events was smaller than the nominal count (clustering-adjusted effective EPV 5.0). Although a participant-level sensitivity analysis produced discrimination that did not differ significantly from the knee-level model, this clustering reduces the effective information available for model estimation, and the findings should therefore be regarded as exploratory pending validation in larger independent cohorts. Replication in larger prospective cohorts with longer follow-up and radiographically confirmed outcomes is required to determine the generalizability of the extensor mechanism mechanical profiling approach.

### Conclusions

In community-dwelling older adults free of knee symptoms at baseline, KESt and PTS were independently and consistently associated with 2-year incident clinical KOA, whereas QMPS was not retained as a prognostic predictor once KESt and PTS were simultaneously accounted for. Risk stratification based on extensor mechanism mechanical properties identified a subgroup with a 24-month KOA incidence more than twice that of the lowest-risk group, suggesting that baseline mechanical profiling of the extensor mechanism may provide clinically meaningful prognostic information prior to symptom onset. These findings support KESt and PTS as candidate targets that warrant further investigation. Given the modest sample, the clustered knee-level structure, and the reduced effective event count, these findings should be interpreted as exploratory and preliminary rather than as support for immediate use of these parameters in screening or preventive intervention. Confirmation in larger independent cohorts with radiographically confirmed outcomes is required before any clinical application.

## Supplementary material

10.2196/84494 Multimedia Appendix 1 Detailed measurement protocols for shear-wave elastography, quadriceps stiffness, patellar tendon stiffness, and knee extensor strength.

10.2196/84494 Multimedia Appendix 2 Hyperparameter tuning grids and selected values for all algorithms.

10.2196/84494 Multimedia Appendix 3 Schematic of the 5-fold nested cross-validation design.

10.2196/84494 Multimedia Appendix 4 Missing data summary for predictor variables (N=220 knees).

10.2196/84494 Multimedia Appendix 5 Spearman correlations among the 3 quadriceps muscle stiffness components.

10.2196/84494 Multimedia Appendix 6 Feature selection history across the 5 outer cross-validation folds.

10.2196/84494 Multimedia Appendix 7 Fold-level C-index variability across the 5 outer cross-validation folds for all 7 algorithms.

10.2196/84494 Multimedia Appendix 8 Permutation test results for model discrimination.

10.2196/84494 Multimedia Appendix 9 Fold-level C-indices and Brier scores across the 5 outer cross-validation folds.

10.2196/84494 Multimedia Appendix 10 Twenty-four–month knee osteoarthritis incidence by gradient boosting survival linear predictor risk tertile.

10.2196/84494 Multimedia Appendix 11 Overall Kaplan-Meier knee osteoarthritis–free survival for the full analytic sample.

10.2196/84494 Multimedia Appendix 12 Sex-stratified C-indices for the gradient boosting survival model.

10.2196/84494 Multimedia Appendix 13 Sex-stratified Kaplan-Meier knee osteoarthritis–free survival and C-index comparison.

## Abbreviations

ACR: American College of Rheumatology
Cox_CR: cluster-robust Cox proportional hazards regression
ElasticNet_Cox: ElasticNet-penalized Cox regression
EPV: events-per-variable
GBS: gradient boosting survival
HR: hazard ratio
ICC: intraclass correlation coefficient
KESt: knee extensor strength
KOA: knee osteoarthritis
LASSO: Least Absolute Shrinkage and Selection Operator
LASSO_Cox: LASSO-penalized Cox regression
LGBS: LASSO gradient boosting survival
MRI: magnetic resonance imaging
PTS: patellar tendon stiffness
QMPS: quadriceps muscle passive stiffness
RSF: random survival forest
SHAP: Shapley Additive Explanations
SurvSVM: survival support vector machine
VAS: Visual Analog Scale
