# Supplementary material for: Prediction of 2-Year Incident Clinical Knee Osteoarthritis From Extensor Mechanism Mechanical Properties in Asymptomatic Community-Dwelling Older Adults: Prospective Cohort Study
Source: JMIR Aging. 2026 Sep 24;9:e84494. doi: 10.2196/84494 (PMC13608216; doi:10.2196/84494)
Supplement: Supplementary file 1 [file aging-v9-e84494-s001.docx]

**Multimedia Appendix 1. Detailed Measurement Protocols**

**S1.1. Shear-Wave Ultrasound Elastography: General Setup**

All shear-wave ultrasound elastography (SWE) measurements were performed using an Aixplorer ultrasound system (Version 4.2; Supersonic Imagine, Aix-en-Provence, France) equipped with a linear transducer (SL 10-2 probe; 2–10 MHz). Room temperature was maintained at 25°C throughout all assessments to standardise passive tissue properties, as ambient temperature is known to influence muscle viscoelastic behaviour (Alfuraih et al., 2019). Participants rested in the measurement position for a minimum of 5 minutes prior to each measurement to allow passive muscle unloading and to ensure a resting steady state. The transducer was applied with minimal contact pressure to avoid artificial stiffness elevation due to tissue compression. Shear modulus (kPa) was calculated using the formula E = 3ρc², where ρ is tissue density (assumed 1000 kg/m³) and c is the propagation velocity of shear waves within the tissue (Zhang & Fu, 2013). All shear modulus values were computed from colour-coded elastogram maps exported from the Aixplorer system using a custom MATLAB script (MathWorks, Inc., Natick, MA, USA).

**S1.2. Quadriceps Muscle Stiffness: Measurement Protocol**

Participants were positioned supine with both knees supported at 60° flexion using a rigid adjustable brace, and hips maintained in neutral rotation. This joint angle was selected because it represents a mid-range position at which passive quadriceps stiffness can be reliably assessed without contributions from active tension or passive insufficiency at either extreme of range (Xu et al., 2021).

Measurement sites for the three superficial quadriceps muscles were standardised as follows. For the rectus femoris (RF), the measurement site was located at the midpoint between the anterior superior iliac spine (ASIS) and the superior border of the patella along the line connecting these two landmarks. For the vastus lateralis (VL), the site was located at the distal one-third of the line connecting the ASIS to the lateral border of the patella. For the vastus medialis (VM), the site was located at the distal one-fifth of the line connecting the ASIS to the medial border of the patella (Li et al., 2023).

At each site, the transducer was positioned longitudinally along the estimated muscle fibre direction with minimal pressure. An 11-second continuous elastography video (acquisition rate: 1 frame/second; spatial resolution: 1×1 mm) was captured at rest for each muscle. The middle five frames of each video were extracted for analysis to reduce variability associated with the initiation and termination of shear wave propagation. For each selected frame, a circular region of interest (diameter: 1.5 cm) was placed centrally within the muscle belly, avoiding fascial boundaries and large echogenic artefacts. The mean shear modulus across the five frames was taken as the representative value for each muscle (Li et al., 2023).

Quadriceps composite stiffness was computed as the unweighted arithmetic mean of the RF, VL, and VM shear modulus values. Unweighted averaging was selected because the three muscles contribute comparably to passive extensor mechanism loading in the resting position assessed, and no prior evidence supports differential weighting. Intra-rater reliability, assessed using a two-week test-retest design, was excellent across all three sites (ICC: RF = 0.94, VL = 0.89, VM = 0.94; Li et al., 2023).

**S1.3. Patellar Tendon Stiffness: Measurement Protocol**

Following quadriceps measurement, participants were repositioned supine with the knee flexed to 30°, supported by a firm foam block placed under the popliteal fossa, and the ankle passively stabilised to prevent involuntary quadriceps contraction. The 30° knee flexion angle was selected to allow the patellar tendon to be assessed in a partially unloaded but passive state, consistent with prior SWE investigations of patellar tendon mechanical properties in aging populations (Hsiao et al., 2015; Zhang & Fu, 2013).

The patellar tendon was identified using B-mode ultrasonography, scanning longitudinally along the anterior aspect of the knee. The tendon was confirmed by its characteristic fibrillar echotexture and bilateral attachment between the inferior pole of the patella and the tibial tuberosity. A rectangular region of interest (13.5 × 12.5 mm) was placed distal to the inferior pole of the patella, entirely within the proximal third of the tendon. Within this rectangle, a circular quantification region was centred approximately 5 mm distal to the patella apex, consistent with established patellar tendon SWE methodology (Zhang et al., 2014; Cook et al., 2000; Kulig et al., 2013). This proximal region was selected because it corresponds to the site of highest mechanical stress concentration during knee extension and the most common location of structural change in aging tendons (Zhang et al., 2014).

Five elastography images were acquired with a minimum of 10 seconds between acquisitions to allow shear wave dissipation. For each image, the mean shear modulus within the circular quantification region was recorded. The arithmetic mean of the five images was taken as the representative patellar tendon stiffness value. Intra-rater reliability was excellent (ICC = 0.98; Zhang & Fu, 2013).

**S1.4. Knee Extensor Strength: Measurement Protocol**

Isokinetic knee extensor peak torque was measured using an isokinetic dynamometer (Cybex Co., Ronkonkoma, NY, USA). Participants were seated with the hip at 85° flexion, the backrest adjusted to maintain this position throughout testing, and stabilisation straps secured across the chest and pelvis to minimise compensatory trunk movement. The dynamometer rotational axis was carefully aligned with the lateral femoral epicondyle by direct palpation before each testing session (Segal et al., 2009).

Following familiarisation, participants performed one warm-up set at approximately 50% maximal voluntary effort. After two minutes of rest, five maximal concentric contractions were performed through the full arc of knee motion (90° flexion to 0° extension) at a constant angular velocity of 60°/s. Verbal encouragement was standardised in content and delivery across participants. The peak torque values from the middle three of the five trials were averaged and normalised to body mass (Nm/kg) to account for inter-individual differences in body size (Segal et al., 2009). Test-retest reliability for this protocol was excellent (ICC = 0.90; Li et al., 2023).
