# Supplementary material for: Prediction of 2-Year Incident Clinical Knee Osteoarthritis From Extensor Mechanism Mechanical Properties in Asymptomatic Community-Dwelling Older Adults: Prospective Cohort Study
Source: JMIR Aging. 2026 Sep 24;9:e84494. doi: 10.2196/84494 (PMC13608216; doi:10.2196/84494)
Supplement: Supplementary file 2 [file aging-v9-e84494-s002.docx]

**Multimedia Appendix 2. Hyperparameter Tuning**

All hyperparameters were optimized within each outer training fold using an inner five-fold cross-validation loop, so that hyperparameter selection was entirely independent of test-fold observations. For LASSO_Cox, the penalty parameter λ was selected automatically by inner cross-validation using the lambda.1se criterion. For ElasticNet_Cox, the mixing parameter α was additionally searched over {0.25, 0.50, 0.75}, with λ selected per α via inner cross-validation, and the α yielding the highest test-fold C-index was retained. For RSF, a grid search was conducted over minimum node size {3, 5, 10}, number of candidate split variables (mtry; {1, √p, p}), and splitting rule {"logrank", "bs.gradient"}, with the combination minimising out-of-bag error selected; the final model was re-fitted with 500 trees. For GBS, a two-dimensional grid search was conducted over the number of boosting iterations (mstop; {50, 100, 200, 500}) and the shrinkage parameter (ν; {0.05, 0.10, 0.50}), selecting the combination maximising inner-fold C-index. For LGBS, the same ν grid was applied, with the optimal mstop for each ν candidate determined via the mboost internal cross-validation function (five-fold, ceiling at 500 iterations), and the ν yielding the highest test-fold C-index was retained. For SurvSVM, the kernel width and regularization parameter γ was searched over {0.001, 0.01, 0.1, 1, 10} using inner five-fold cross-validation with C-index as the selection criterion.
