# Supplementary material for: Prediction of 2-Year Incident Clinical Knee Osteoarthritis From Extensor Mechanism Mechanical Properties in Asymptomatic Community-Dwelling Older Adults: Prospective Cohort Study
Source: JMIR Aging. 2026 Sep 24;9:e84494. doi: 10.2196/84494 (PMC13608216; doi:10.2196/84494)
Supplement: Supplementary file 3 [file aging-v9-e84494-s003.docx]

## **Multimedia Appendix 3. Schematic of the five-fold nested cross-validation design.**

Illustration of the participant-stratified five-fold nested cross-validation framework. In each outer fold, the training partition was used for (i) LASSO-penalized inner cross-validation for feature selection (lambda.1se criterion) and (ii) hyperparameter tuning via dedicated inner five-fold cross-validation for each algorithm. The test fold was held out entirely from both steps. Folds were stratified at the participant level by event status to prevent data leakage from bilateral knee observations. Each outer fold contributed one C-index estimate; the five fold-level estimates were aggregated as the mean (± SD) C-index. LASSO, least absolute shrinkage and selection operator.
