# Supplementary material for: Prediction of 2-Year Incident Clinical Knee Osteoarthritis From Extensor Mechanism Mechanical Properties in Asymptomatic Community-Dwelling Older Adults: Prospective Cohort Study
Source: JMIR Aging. 2026 Sep 24;9:e84494. doi: 10.2196/84494 (PMC13608216; doi:10.2196/84494)
Supplement: Supplementary file 4 [file aging-v9-e84494-s004.docx]

## **Multimedia Appendix 4. Missing data summary for predictor variables (n = 220 knees).**

| **Variable** | **Missing (%)** | **N missing** | **N total** |
| --- | --- | --- | --- |
| Patellar tendon stiffness | 2.73% | 6 | 220 |
| Rectus femoris stiffness | 1.36% | 3 | 220 |
| Knee flexion strength | 0.45% | 1 | 220 |
| Vastus lateralis stiffness | 0.45% | 1 | 220 |
| All other variables | 0% | 0 | 220 |

Variables with no missing data are grouped in the last row. Multiple imputation was performed using MICE (m = 20 datasets, predictive mean matching, 50 iterations). All remaining variables (age, sex, BMI, vastus medialis stiffness, hip abduction strength) had 0% missing.
