# Supplementary material for: Prediction of 2-Year Incident Clinical Knee Osteoarthritis From Extensor Mechanism Mechanical Properties in Asymptomatic Community-Dwelling Older Adults: Prospective Cohort Study
Source: JMIR Aging. 2026 Sep 24;9:e84494. doi: 10.2196/84494 (PMC13608216; doi:10.2196/84494)
Supplement: Supplementary file 5 [file aging-v9-e84494-s005.docx]

## **Multimedia Appendix 5. Spearman correlations among the three quadriceps muscle stiffness components.**

| **Component pair** | **Spearman r** |
| --- | --- |
| Rectus femoris – Vastus lateralis | 0.402 |
| Rectus femoris – Vastus medialis | 0.472 |
| Vastus lateralis – Vastus medialis | 0.344 |

Correlations computed on complete cases (n = 217 knees). Moderate inter-component correlations (r = 0.344–0.472) supported aggregation into a single quadriceps composite stiffness (QMPS) variable, calculated as the mean of rectus femoris, vastus lateralis, and vastus medialis shear modulus values.
