# Supplementary material for: Prediction of 2-Year Incident Clinical Knee Osteoarthritis From Extensor Mechanism Mechanical Properties in Asymptomatic Community-Dwelling Older Adults: Prospective Cohort Study
Source: JMIR Aging. 2026 Sep 24;9:e84494. doi: 10.2196/84494 (PMC13608216; doi:10.2196/84494)
Supplement: Supplementary file 6 [file aging-v9-e84494-s006.docx]

## **Multimedia Appendix 6. Feature selection history across five outer cross-validation folds.**

| **Fold** | **N train** | **N test** | **Events (train)** | **Events (test)** | **N selected** | **EPV** | **Selected predictors** |
| --- | --- | --- | --- | --- | --- | --- | --- |
| 1 | 174 | 46 | 44 | 11 | 2 | 22.0 | PTS, KESt |
| 2 | 177 | 43 | 44 | 11 | 2 | 22.0 | PTS, KESt |
| 3 | 176 | 44 | 43 | 12 | 2 | 21.5 | PTS, KESt |
| 4 | 176 | 44 | 43 | 12 | 4 | 10.8 | Age, Sex, PTS, KESt |
| 5 | 177 | 43 | 46 | 9 | 2 | 23.0 | PTS, KESt |

Feature selection was performed via inner five-fold LASSO-penalized Cox regression (lambda.1se) independently within each outer training fold. Patellar tendon stiffness (PTS) and knee extensor strength (KESt) were selected in all five folds. In Fold 4, age and sex were additionally selected. Quadriceps composite stiffness was never selected across any fold. EPV, events per variable.
