# Supplementary material for: Prediction of 2-Year Incident Clinical Knee Osteoarthritis From Extensor Mechanism Mechanical Properties in Asymptomatic Community-Dwelling Older Adults: Prospective Cohort Study
Source: JMIR Aging. 2026 Sep 24;9:e84494. doi: 10.2196/84494 (PMC13608216; doi:10.2196/84494)
Supplement: Supplementary file 7 [file aging-v9-e84494-s007.docx]

## **Multimedia Appendix 7. Fold-level C-index variability across five outer cross-validation folds for all seven algorithms.**

Each panel shows the C-index distribution across five outer folds for one algorithm. Fold-level C-indices are shown as individual points connected by lines; the horizontal dashed line indicates the fold-mean C-index. RSF exhibited the greatest fold-level variability (SD = 0.185, range: 0.348–0.818), substantially exceeding all other algorithms (SD range: 0.037–0.060). GBS and LGBS showed the most consistent fold-level performance. Cox_CR, cluster-robust Cox proportional hazards regression; LASSO_Cox, L1-penalized Cox; ElasticNet_Cox, L1+L2-penalized Cox; RSF, Random Survival Forest; GBS, Gradient Boosting Survival; LGBS, ComponentwiseLasso Gradient Boosting Survival; SurvSVM, Survival Support Vector Machine.
