# Supplementary material for: Prediction of 2-Year Incident Clinical Knee Osteoarthritis From Extensor Mechanism Mechanical Properties in Asymptomatic Community-Dwelling Older Adults: Prospective Cohort Study
Source: JMIR Aging. 2026 Sep 24;9:e84494. doi: 10.2196/84494 (PMC13608216; doi:10.2196/84494)
Supplement: Supplementary file 8 [file aging-v9-e84494-s008.docx]

**Multimedia Appendix 8. Permutation test results for model discrimination.**

| **Algorithm** | **Observed C** | **Null mean C** | **Null SD** | **p-value** | **N permutations** |
| --- | --- | --- | --- | --- | --- |
| GBS (best) | 0.720 | 0.529 | 0.073 | < 0.0101 | 999 |
| Cox_CR (reference) | 0.715 | 0.532 | 0.076 | < 0.0101 | 999 |

Participant-level outcome label shuffling was applied to preserve the bilateral-knee structure (999 permutations). The empirical p-value is the proportion of permuted C-indices meeting or exceeding the observed value. The minimum achievable p-value with 999 permutations is p < 0.0101.
