# Supplementary material for: Prediction of 2-Year Incident Clinical Knee Osteoarthritis From Extensor Mechanism Mechanical Properties in Asymptomatic Community-Dwelling Older Adults: Prospective Cohort Study
Source: JMIR Aging. 2026 Sep 24;9:e84494. doi: 10.2196/84494 (PMC13608216; doi:10.2196/84494)
Supplement: Supplementary file 9 [file aging-v9-e84494-s009.docx]

## **Multimedia Appendix 9. Fold-level C-indices and Brier score across five outer cross-validation folds.**

| **Fold** | **Cox_CR** | **LASSO** | **ElasticNet** | **RSF** | **GBS** | **LGBS** | **SurvSVM** | **BS†** |
| --- | --- | --- | --- | --- | --- | --- | --- | --- |
| 1 | 0.739 | 0.723 | 0.723 | 0.818 | 0.743 | 0.743 | 0.435 | 0.061 |
| 2 | 0.751 | 0.714 | 0.731 | 0.771 | 0.754 | 0.754 | 0.369 | 0.017 |
| 3 | 0.740 | 0.736 | 0.732 | 0.706 | 0.740 | 0.740 | 0.336 | 0.052 |
| 4 | 0.681 | 0.668 | 0.665 | 0.684 | 0.699 | 0.684 | 0.460 | 0.152 |
| 5 | 0.662 | 0.647 | 0.657 | 0.348 | 0.662 | 0.667 | 0.475 | 0.013 |

† Inverse probability of censoring-weighted Brier score at 24 months (GBS model). Substantial variability in RSF C-index (range: 0.348–0.818) reflects sensitivity to fold-specific event distributions in the limited sample. Cox_CR, cluster-robust Cox; GBS, Gradient Boosting Survival; LGBS, ComponentwiseLasso GBS; RSF, Random Survival Forest; BS, Brier score.
