# Supplementary material for: Prediction of 2-Year Incident Clinical Knee Osteoarthritis From Extensor Mechanism Mechanical Properties in Asymptomatic Community-Dwelling Older Adults: Prospective Cohort Study
Source: JMIR Aging. 2026 Sep 24;9:e84494. doi: 10.2196/84494 (PMC13608216; doi:10.2196/84494)
Supplement: Supplementary file 10 [file aging-v9-e84494-s010.docx]

## **Multimedia Appendix 10. Twenty-four-month KOA incidence by GBS linear predictor risk tertile.**

| **Risk tertile** | **N**  **knees** | **N**  **participants** | **N**  **events** | **Event rate (%)** | **24-month KM incidence**  **(95% CI)** |
| --- | --- | --- | --- | --- | --- |
| Low | 74 | 47 | 13 | 17.6% | 0.449 (0.097–0.664) |
| Intermediate | 73 | 57 | 14 | 19.2% | 0.411 (0.105–0.612) |
| High | 73 | 48 | 28 | 38.4% | 0.578 (0.371–0.717) |

Risk tertiles were defined by GBS linear predictor score. The high-risk tertile had more than twice the event rate of the low-risk tertile (38.4% vs. 17.6%). KM, Kaplan-Meier; KOA, knee osteoarthritis.
