# Supplementary material for: Prediction of 2-Year Incident Clinical Knee Osteoarthritis From Extensor Mechanism Mechanical Properties in Asymptomatic Community-Dwelling Older Adults: Prospective Cohort Study
Source: JMIR Aging. 2026 Sep 24;9:e84494. doi: 10.2196/84494 (PMC13608216; doi:10.2196/84494)
Supplement: Supplementary file 11 [file aging-v9-e84494-s011.docx]

## **Multimedia Appendix 11. Overall Kaplan-Meier KOA-free survival for the full analytic sample.**

Kaplan-Meier KOA-free survival probability over 24 months for the full analytic sample (n = 220 knees, 116 participants; 55 events, 25.0%). The shaded area represents the 95% confidence interval. The risk table below the curve indicates the number of knees remaining at risk at each time point. KOA, knee osteoarthritis.
