# Supplementary material for: Prediction of 2-Year Incident Clinical Knee Osteoarthritis From Extensor Mechanism Mechanical Properties in Asymptomatic Community-Dwelling Older Adults: Prospective Cohort Study
Source: JMIR Aging. 2026 Sep 24;9:e84494. doi: 10.2196/84494 (PMC13608216; doi:10.2196/84494)
Supplement: Supplementary file 12 [file aging-v9-e84494-s012.docx]

## **Multimedia Appendix 12. Sex-stratified C-indices for the GBS model.**

| **Subgroup** | **N knees** | **N events** | **C-index** | **95% CI lower** | **95% CI upper** | **Interaction p** |
| --- | --- | --- | --- | --- | --- | --- |
| Male | 83 | 20 | 0.727 | 0.564 | 0.835 | — |
| Female | 137 | 35 | 0.748 | 0.663 | 0.826 | — |
| Overall | 220 | 55 | 0.705 | 0.629 | 0.778 | 0.485 |

Bootstrap 95% CI derived from 500 resamples. The interaction p-value is from a sex-by-predictor interaction term in a cluster-robust Cox model. The non-significant interaction (p = 0.485) indicates no meaningful effect modification by sex. CI, confidence interval.
