# Supplementary material for: Prediction of 2-Year Incident Clinical Knee Osteoarthritis From Extensor Mechanism Mechanical Properties in Asymptomatic Community-Dwelling Older Adults: Prospective Cohort Study
Source: JMIR Aging. 2026 Sep 24;9:e84494. doi: 10.2196/84494 (PMC13608216; doi:10.2196/84494)
Supplement: Supplementary file 13 [file aging-v9-e84494-s013.docx]

## **Multimedia Appendix 13. Sex-stratified Kaplan-Meier KOA-free survival and C-index comparison.**

(A) Kaplan-Meier KOA-free survival curves stratified by sex. Male subgroup: n = 83 knees, 20 events (24.1%); female subgroup: n = 137 knees, 35 events (25.5%). Shaded areas represent 95% confidence intervals. (B) GBS C-indices with bootstrap 95% confidence intervals (500 resamples) for male (C = 0.727, 95% CI: 0.564–0.835), female (C = 0.748, 95% CI: 0.663–0.826), and overall (C = 0.705, 95% CI: 0.629–0.778) subgroups. A formal sex-by-predictor interaction term in a cluster-robust Cox model was non-significant (p = 0.485), indicating no meaningful effect modification by sex. KOA, knee osteoarthritis; GBS, Gradient Boosting Survival; CI, confidence interval.
